# TetRex: a novel algorithm for index-accelerated search of highly conserved motifs

**DOI:** 10.1093/nargab/lqaf039

**Published:** 2025-04-17

**Authors:** Remy M Schwab, Simon Gene Gottlieb, Knut Reinert

**Affiliations:** Max-Planck-Institute for Molecular Genetics, Ihnestrasse 63, 14195 Berlin, Germany; Algorithmic Bioinformatics, Freie Universität Berlin, Takustrasse 9, 14195 Berlin, Germany; Algorithmic Bioinformatics, Freie Universität Berlin, Takustrasse 9, 14195 Berlin, Germany; Max-Planck-Institute for Molecular Genetics, Ihnestrasse 63, 14195 Berlin, Germany; Algorithmic Bioinformatics, Freie Universität Berlin, Takustrasse 9, 14195 Berlin, Germany

## Abstract

The scale of modern datasets has necessitated innovations to solve even the most traditional and fundamental of computational problems. Set membership and set cardinality are both examples of simple queries that, for large enough datasets, quickly become challenging using traditional approaches. Interestingly, we find a need for these innovations within the field of biology. Despite the vast differences among living organisms, there exist functions so critical to life that they are conserved in the genomes and proteomes across seemingly unrelated species. Regular expressions (regexes) can serve as a convenient way to represent these conserved sequences compactly. However, despite the strong theoretical foundation and maturity of tools available, the state-of-the-art regex search falls short of what is necessary to quickly scan an enormous database of biological sequences. In this work, we describe a novel algorithm for regex search that reduces the search space by leveraging a recently developed compact data structure for set membership, the hierarchical interleaved bloom filter. We show that the runtime of our method combined with a traditional search outperforms state-of-the-art tools.

## Introduction

Regular Expressions (RegExes) are a powerful and ubiquitous method to represent a regular language succinctly. Their theoretical foundation in automata theory provides not only a means for concisely representing textual patterns but also the algorithms and data structures to search for instances matching them. Initial, practical uses of regular expressions (regexes) emerged in search engines, compilers, and web form validation. Today, rapid digitalization of medical and biochemical data has made regular expressions (regexes) also a useful tool for bioinformatics. However, while much research and development has been devoted to making the underlying RegEx engine fast or able to match complex patterns, comparatively less work has been done on leveraging indexes. Hence, the runtime of most state-of-the-art approaches is still at least linear in the size of the text, a limitation that becomes particularly prohibitive within the context of bioinformatics, where some advanced analysis of biological sequences requires the use of sophisticated query patterns and sequence profiles. Since DNA and proteins can be seen as regular languages, this calls for query models that support the definition of regexes.

For instance, such regex queries enable conclusions on the significance of protein patterns and profiles, e.g. based on the PROSITE collection [1]. Similarly, the JASPAR database [2] contains DNA profiles of transcription factor binding sites, which are identified by standard (quadratic time) local alignment algorithms with position-specific costs or profile Hidden Markov Models (pHMMs).

**Figure 1.**
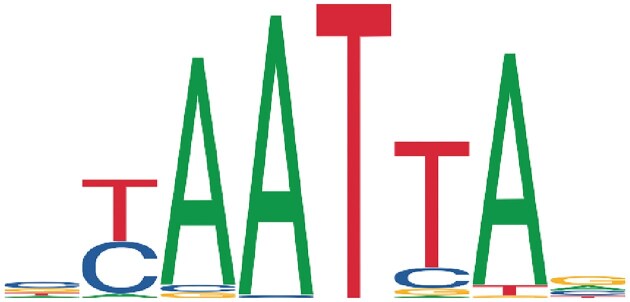
Sequence logo of the class of human homeodomain factors from JASPAR, which can be written as regex [TC]-A-A-T-[TC]-[AT].

**Figure 2.**
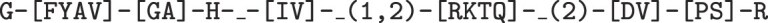
Regex of the citrate synthase pattern from PROSITE, including disjunctions of amino acids, wildcards (denoted as _), and constraints on the number of occurrences [such as (1,2)].

As an example, consider the profile of the class of human homeodomain factors from JASPAR, which is illustrated as a sequence logo in Fig. 1. To query for an occurrence of this pattern, the regex in the caption includes disjunctions of nucleotides for certain positions to detect probable instantiations of the pattern. Querying protein patterns, in turn, requires more expressive regex queries. Figure 2 gives the example of the citrate synthase pattern from PROSITE. It features disjunctions of amino acids, wildcards, and constraints on the number of occurrences. In the general case, regex queries may further include Kleene closure and variables to capture the repeated occurrence of specific subsequences.

Accelerating the search for regexes has seen previous attention. From 2002 to 2011, a series of works seemed to culminate in the LPMS algorithm by Tsang and Chawla [3]. In their work, the authors presented an integer linear program to optimize their choice of so-called multigrams, i.e. *k*-mers with different values of *k*. This use of multigrams stems from the FREE model introduced by Cho and Rajagopalan [4]. In the FREE model, the objective is to select the minimal useful multigram set. A multigram is considered useful if the fraction of documents containing the multigrams is smaller than a threshold. Otherwise, it is considered ‘useless’.

A limitation of the FREE approach is that it did not consider possible queries during indexing and thus many queries ended up not utilizing the index at all. To overcome this, Hore *et al.* [5] proposed a multigram selection algorithm called BEST, which considers both the database and query workload. In the BEST algorithm, each multigram has an associated cost *c* and benefit *b*. The benefit of the multigram is derived from the number of records that can be pruned when utilized by a query. The ratio of *b*/*c* forms the utility value, which is then used as the objective function to optimize both the index efficiency and query hit rate at the same time.

The LPMS method used combinatorial optimization to select a set of prefix-free multigrams for indexing. Their method guaranteed that the running time of a query using the index is less than the full database scan and even evaluated the search of PROSITE [1] patterns as a practical application of their method. Unfortunately, despite the promising potential of the LPMS approach, the paper provides no details of the absolute runtime. Instead, they report only precision and recall. Furthermore, none of the above-cited works have an implementation freely available (despite our efforts to obtain them).

Seemingly in parallel with the development of the aforementioned methods, Cox *et al.* developed the Google Code Search engine (see https://swtch.com/∼rsc/regexp/regexp4.html), which was launched on 5 October 2006. Code search enabled users to quickly search all publicly available source code. Notably, it utilized a trigram (*k*= 3), inverted index over documents, and allowed for precise queries using RegExes by decomposing them into their constituent trigrams. The documents identified to contain putative matches could be subsequently verified with a full RegEx search, thereby avoiding a search across the entire database of documents. Code search was discontinued on 15 January 2012, but a (simplified) working implementation of it remains hosted on GitHub (https://github.com/google/codesearch).

Interestingly, a 2022 paper from Qiu *et al.* [6] proposed a method called Treematch, which utilized an inverted index over documents. By making use of the positional information of the *k*-mers, they devised an algorithm similar to the seed-chain-extend approach used by the popular read aligner minimap2 [7]. For a given RegEx, they construct a so-called ‘gram-driven’ nondeterministic finite automaton (NFA) in which the transitions between states are *k*-mers/ngrams. As they traverse this gram-driven NFA, the *k*-mers of the NFA are looked up in the index. Since any match of a RegEx must occur exactly in the text, they were able to bypass the verification step by chaining together *k*-mers that occur one position apart from each other within the same document. While this seems like an exciting and promising approach, upon obtaining this software, we found it to be unsuitable for comparison due to hard-coded filepaths, poor documentation, and undocumented, incomprehensible input file formatting requirements. Despite numerous communications with the corresponding author, we were unable to reproduce the results of the paper.

In addition to the general-purpose RegEx methods mentioned, there are also some tools available for the querying of biologically meaningful RegEx. However, they are largely of heuristic nature and neither guarantee to find all matches nor model true regexes, like PHI-Blast, or they take seconds for one query on a small database like the PROSITE scan tool [8].

We emphasize that, in their 2021 article, Gibney and Thankachan [9] showed that the general problem of indexing a text for answering regex queries is difficult. They formally proved that, regardless of the extent to which the text is preprocessed, regex queries cannot be answered in strongly sublinear time. However, despite the algorithmic lower bounds, we hypothesize that by indexing the *k*-mers of the text and first relaxing the problem of locating matches to determining which portions of the text might contain a match, we can at least solve this problem efficiently for practical purposes.

Inspired by recent advances in storing and querying large sets of *k*-mers, we (i) propose an algorithm to construct an acyclic graph *G*^*k*^ representing all necessary *k*-mers for matching any word represented by a RegEx, (ii) propose an algorithm that rapidly narrows the search space by traversing *G*^*k*^ and simultaneously interfacing with the index, and (iii) provide an efficient command-line tool implementing these algorithms that enables users to work with common biological data types.

## Materials and methods

In this section, we provide the necessary background as well as give a general overview of our method.

### RegExes, NFAs, and *k*-mers

We start by defining regexes in the form we accept them. For convenience, we include the + operator, although not strictly necessary. Other shorthand operators are omitted for simplicity.

Definition 1.

- ∅ is a regular expression. It denotes the *empty language*. *L*(∅) = ∅.
- ϵ is a regular expression. Its language consists of the *empty word*. *L*(ϵ) = {ϵ}.
- For each *single letter*, *a* ∈ Σ, *a* is a regular expression. *L*(*a*) = {*a*}.
- Let *R*_1_ and *R*_2_ be regexes. Then, we can use the operators {○, |, *, +} to define the following regexes:
  \begin{eqnarray*}
{{\rm product}} \;\;\; (R_1\circ R_2) \;\; &&{\rm with} \qquad \quad L(R_1R_2) := L(R_1)L(R_2),\\ {{\rm union}} \;\; ( R_1 \mid R_2 ) \;\; &&{\rm with} \quad L(( R_1 \mid R_2 )) := L(R_1) \cup L(R_2),\\ {{\rm star}} \;\; (R)^{*} \;\;\;\;\, &&{\rm with} \qquad \quad L((R)^*) := (L(R))^*,\\ {{\rm plus}} \;\; (R)^{+} \;\;\;\;\, && {\rm with} \qquad \quad L((R)^{+}) := L(R)(L(R))^{*}
.\end{eqnarray*}

Note that in our examples, we sometimes omit the ○ operator by simply juxtaposing or omitting brackets, assuming the natural operator preference. Since we will use *k*-mers for filtering, we assume as one condition for our method that *L*(*R*) does not contain words shorter than *k*. Also, we assume that subexpressions in a * or + expression do not contain the empty word.

### Indexing the database

In the context of bioinformatics, a database of sequences often simply refers to a large, FASTA-formatted text file containing many sequence records of biological origin. For our purposes, we split this one large file into *u* files called ‘bins’. To index such a database, any data structure that allows efficient querying of *k*-mers across many user bins is feasible. However, the author’s group has recently developed such data structures, called the interleaved bloom filter (IBF) and hierarchical IBF (HIBF) [10]. Indexing of the *u* bins consists of the following steps:

- The bloom filters corresponding to the bins are initialized with all zeroes.
- The constituent sequences of each bin are read in turn.
- Each sequence is decomposed into *k*-mers.
- Each *k*-mer is processed by some user-specified number of hash functions.
- The bits at the corresponding indices of the hash values within the bloom filter for the bin are flipped.

**Figure 3.**
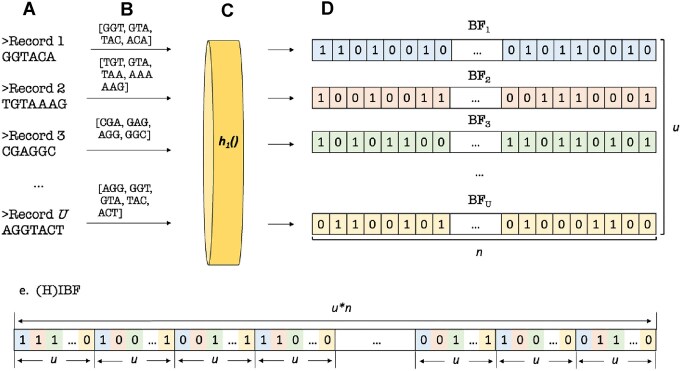
A simplified indexing example where *k*= 3, *u* bins, one hash function (more are possible). (**A**) A database of FASTA-formatted sequences that will be split into *u* bins with one sequence per bin. (**B**) All the 3-mers from each sequence. (**C**) A single hash function to digest *k*-mers (usually three or four are used in practice). (**D**) IBF corresponding to each bin. (**E**) The resulting index with the individual cells of the bloom filters interleaved and each binning bitvector demarcated.

This process is illustrated in Fig. 3. TetRex allows for the indexing of sets of DNA and of protein sequences.

The HIBF is actually an extension of a previous data structure, the IBF [11]. TetRex allows users to choose whether they would like to index their database using the IBF or HIBF. If the database is split into bins of varying size or there are a large number of bins (>1024), we recommend using the HIBF. Otherwise, the IBF generally has a smaller memory footprint.

### Querying the database

Dividing our text into bins (that often coincide with individual files) allows us to quickly test whether a user bin contains all *k*-mers of a word in *L*(*R*).

**Figure 4.**
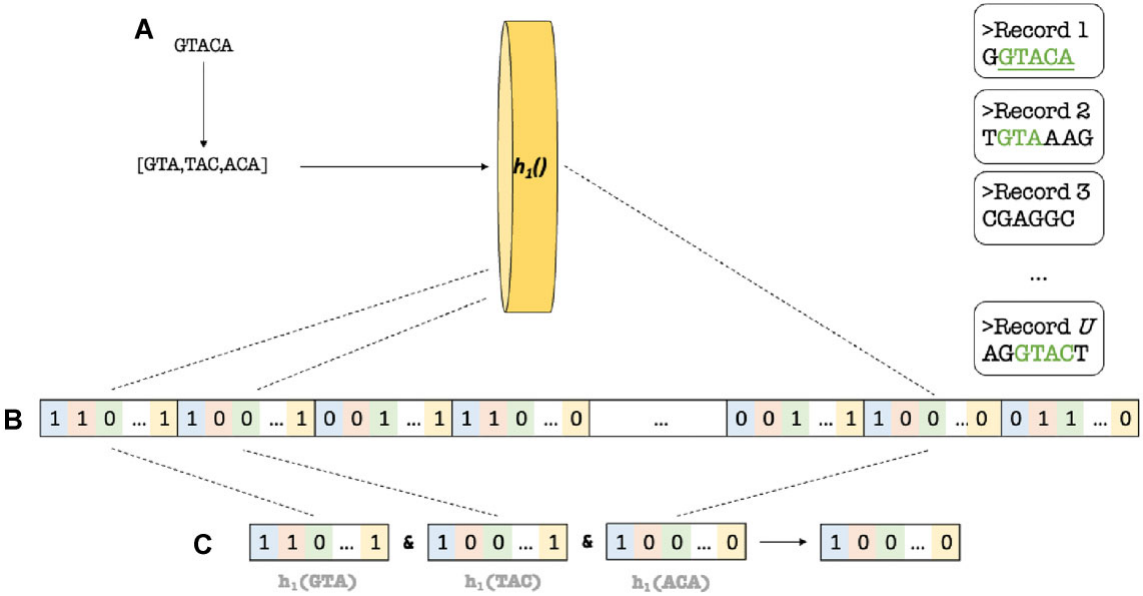
A small querying example using the previous IBF. (**A**) A query composed of three 3-mers. (**B**) Each 3-mer is hashed. (**C**) The resulting bitvectors are bitwise AND’d resulting in just a single bin possibly containing a match.

The (H)IBF enables the rapid query of many bins for the presence of a *k*-mer by computing only a handful of hash functions. It returns a binning bitvector, which is a bitvector for the *u* bins that indicates the membership of a *k*-mer in the respective user bin with a 1 (*positive bin*) and absence with a 0. The process of querying an (H)IBF is illustrated in Fig. 4. For each possible instance of a regex, we can use those binning bitvectors to simultaneously, for all *u* bins, maintain a bitwise AND for the set of all *k*-mers that make up an instance of a RegEx query. However, it is important to note that since RegExes are not linear sequences and contain disjunctions, it is possible for bins that do not share all the same *k*-mers to still contain potential matches. Therefore, matching RegExes with the (H)IBF must also perform bitwise *OR* for the results of all possible instances. This is expanded on in the section on computing positive bins.

### False positives

The bloom filters of the HIBF are a potential source of false positives. This can negatively impact the runtime due to spurious hits that require unnecessary linear verification. Additionally, for a large dataset, a very small false-positive rate can lead to very large index sizes. However, the bloom filter false-positive rate can be specified by the user during the construction of the index for their particular needs and available resources. Furthermore, while a single *k*-mer might be falsely reported with probability *p*, in order for a spurious hit to occur, all *k*-mers of a word would have to be false positives. Therefore, the effective false-positive rate of a query is dictated more by the number of *k*-mers it contains.

An additional source of false positives comes from the loss of coherency from decomposing reference sequences into *k*-mers and not maintaining positional information. Let us have a look at a small example of this idea.

**Example 1**. Assume that we want to match a regex *R* = *A*(*A*|*C*|*G*)(*C*|*G*)*C*(*T*)*A in four user bins $\mathit {UB}_1=\lbrace ACCTTA\rbrace$, $\mathit {UB}_2=\lbrace \underline{ACCCA}AGCC\rbrace$, $\mathit {UB}_3=\lbrace ACC\underline{AGGCTA}\rbrace$, and $\mathit {UB}_4=\lbrace AAGCCA\rbrace$. A short inspection shows that *R* occurs in $\mathit {UB}_2$ as $w$_2_ = *ACCCA* and $\mathit {UB}_3$ as $w$_3_ = *AGGCTA* (shown underlined); hence, $\mathit {UB}_2$ and $\mathit {UB}_3$ are positive bins. A 3-mer based filter, however, would yield three positive results where one is a false positive. $\mathit {UB}_2$ contains {*ACC*, *CCC*, *CCA*} and $\mathit {UB}_3$ contains {*AGG*, *GGC*, *GCT*, *CTA*}. As a false positive, $\mathit {UB}_4$ contains {*AAG*, *AGC*, *GCC*, *CCA*}, which are all 3-mers that make up one possible word in *L*(*R*); however, it does not match *R*.

### Constructing the *k*-graph

In this section, we describe how to construct the graphs *G* and *G*^*k*^, respectively. Although not constructed by the TetRex algorithm, the graph *G* can be seen as an NFA that accepts *L*(*R*). The correctness of this claim follows by observing that the construction of *G* is a node-based version of the NFA construction proposed by Thompson [12]. The graph *G*^*k*^ accepts only a subset of *L*(*R*). However, critically, we note that while *L*(*R*) can be infinitely large, the set of *k*-mers contained in *L*(*R*) is finite. We will show that *G*^*k*^ represents all the necessary *k*-mers to match any word in *L*(*R*). For the construction, we use pairs of nodes (*a*, *b*) that describe a subgraph of *G* with *a* being the *entry* node and *b* being the *exit* node. Given a regex *R* and *k*, we first convert *R* into postfix notation using the well-established shunting-yard algorithm [13]. We then parse the postfix expression and construct nodes and edges of *G* (resp. *G*^*k*^). For this, we use a stack containing pairs of nodes. Each pair contains an entry and an exit node of a subgraph representing a subexpression. Whenever, we parse an operator *op* with *op* ∈ {○, ∣, *, +}, we construct an operator-specific subgraph using the elements from the stack and then push a new pair of nodes onto the stack. The algorithm stops once *R* is parsed with the stack containing a single pair of nodes.

More specifically, let *R* ∈ (Σ∪{○, ∣, *, +})* be a regex in postfix notation over the alphabet Σ. Let *D* = (*V*, *A*) be a digraph. *V* = {*s*, *t*}∪*C*∪*B*∪*J* consists of *character nodes C, split nodes B*, and *join nodes J*. Each character node *spells* a character *c* ∈ Σ and has exactly one outgoing edge. Split nodes and join nodes do not spell any characters. Split nodes can have multiple outgoing edges. Join nodes have several incoming and at most one outgoing edge. *s* and *t* are unique entry and exit nodes.

The algorithm maintains a stack *S* containing pairs of nodes. *R* is parsed left to right and the following operations are performed depending on the parsed character or operand *x*. We illustrate the construction of *G*^*k*^ using the RegEx *R* = TCG*A and *k*= 3 in Fig. 6.

**Figure 5.**
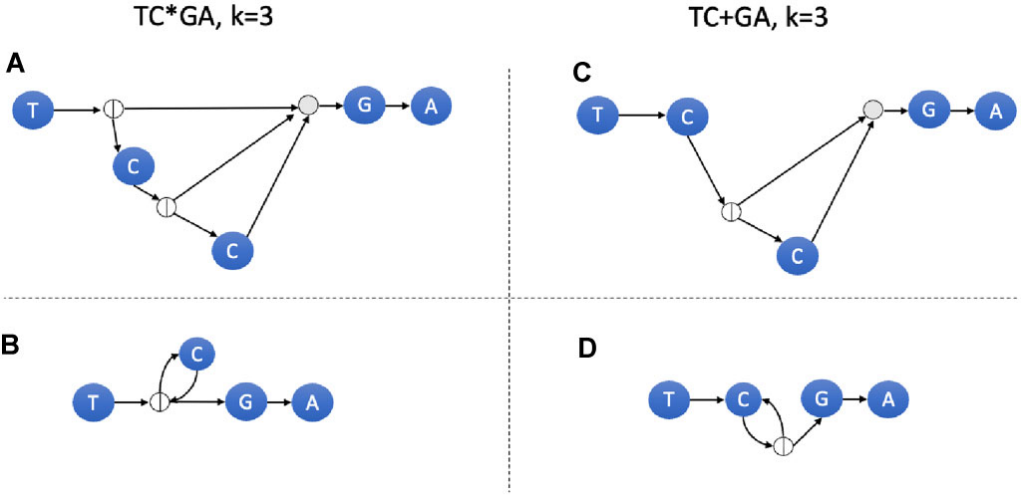
(**A**) *G*^*k*^ construction for Kleene star operator. (**B**) *G* construction for Kleene star. (**C**) *G*^*k*^ construction using + operator. (**D**) *G* construction using + operator.

- *x* ∈ Σ (for *G* and *G*^*k*^):
  We create a character node $v$ spelling *x* and push ($v$, $v$) on *S*.
- *x* = ∣ (for *G* and *G*^*k*^):
  We pop (*a*, *b*) and then (*c*, *d*) from the stack. We create a split node *s*, and a join node *e* in *V*. We add arcs *s* → *a*, *s* → *c*, *b* → *e* and *d* → *e* to *A* and then push (*s*, *e*) to *S*.
- *x* = ○ (for *G* and *G*^*k*^):
  We pop (*a*, *b*) and then (*c*, *d*) from the stack. We add the arc *d* → *a* to *A* and then push (*c*, *b*) to *S*.
- *x* = * (for *G*) (see Fig. 5B):
  We pop (*a*, *b*) from the stack. We create a split node *s* in *V*. We add arcs *s* → *a*, *b* → *s* to *A* and then push (*s*, *s*) to *S*.
- *x* = * (for *G*^*k*^) (see Fig. 5A):
  We pop (*a*, *b*) from the stack. We create *k* − 1 copies (*a*_*i*_, *b*_*i*_) for 1 ≤ *i* ≤ *k* − 1 of the subgraph with entry node *a* and exit node *b*. We create *k* − 1 split nodes *s*_1_, …, *s*_*k* − 1_, and a join node *e* in *V*. We add arcs *s*_*i*_ → *a*_*i*_, *b*_*i*_ → *s*_*i* + 1_ for 1 ≤ *i* ≤ *k* − 2, and *s*_*i*_ → *e* for 1 ≤ *i* ≤ *k* − 2. Also add *b*_*i*_ → *e* and then push (*s*_1_, *e*) to *S*.
- *x* = + (for *G*) (see Fig. 5D):
  We pop (*a*, *b*) from the stack. We create a split node *s* in *V*. We add arcs *s* → *a*, and *b* → *s* to *A* and then push (*a*, *s*) to *S*.
- *x* = + (for *G*^*k*^) (see Fig. 5C):
  We pop (*a*, *b*) from the stack. We create *k* − 1 copies (*a*_*i*_, *b*_*i*_) for 1 ≤ *i* ≤ *k* − 1 of the subgraph with entry node *a* and exit node *b*. We create *k* − 2 split nodes *s*_2_, …, *s*_*k* − 1_, and a join node *e* in *V*. We add arcs *s*_*i*_ → *a*_*i*_ for 2 ≤ *i* ≤ *k* − 2, *b*_*i*_ → *s*_*i* + 1_ for 1 ≤ *i* ≤ *k* − 2, and *s*_*i*_ → *e* for 2 ≤ *i* ≤ *k* − 2. Also add *b*_*i*_ → *e* to *A* and then push (*a*_1_, *e*) to *S*.

**Figure 6.**
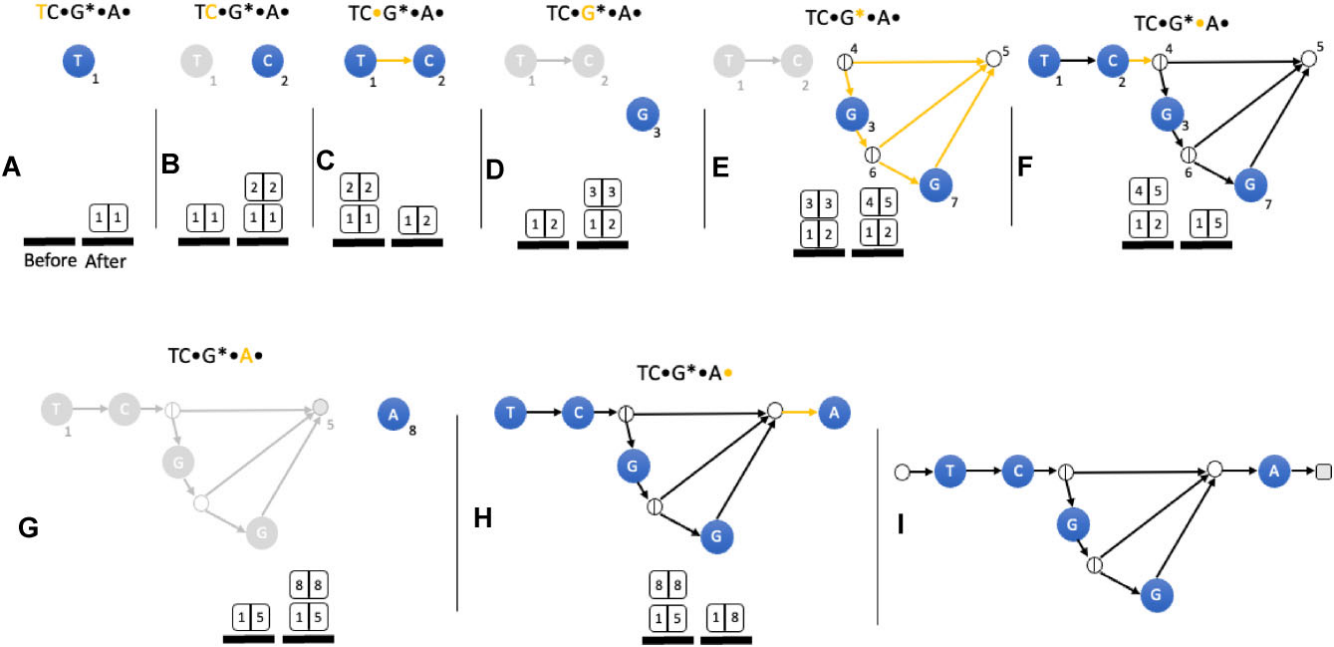
Construction of *G*^*k*^ for *k* = 3. We first transform *R* = TCG*A into postfix notation *TC*○G*○*A*○. (**A**) A character node $v$ = 1 spelling *T* is added to the graph and the node pair (1, 1) is pushed on the stack. (**B**) Another character node $v$ = 2 spelling *C* is added to the graph and (2, 2) is pushed onto the stack. (**C**) Operator ○ triggers two pop operations from the stack. (*a*, *b*) = (2, 2) is the result of the first pop and (*c*, *d*) = (1, 1) the result of the second pop. We then create an arc (*d*, *a*) = (1, 2) and push (*c*, *b*) = (1, 2) onto the stack. (**D**) A character node $v$ = 3 spelling *G* is added and (3, 3) is pushed onto the stack. (**E**) A Kleene star operator triggers one pop of (*a*, *b*) = (3, 3) off the stack. Here the constructions for *G* and *G*^*k*^ differ. For *G*^*k*^ we create a subgraph with *k* − 1 occurrences of the (3, 3) subgraph and place them between a split node *s* = 4 and a join node *e* = 5 in the graph. Then (*s*, *e*) = (4, 5) is pushed onto the stack, allowing to traverse (3, 3) 0, 1, or 2 times. For *G*, we would add a cycle to the graph allowing to traverse (3, 3) infinitely often. (**F**) A concat operator ∣ forms an arc between two subgraphs, (4, 5) and (1, 2) are popped, and (1, 5) is pushed as a new entry, exit pair. (**G**) A character node $v$ = 8 spelling *A* is created. (**H**) The previous character node is concatenated with the larger subgraph. (**I**) The graph is completed by adding the entry and exit nodes *s* and *t*.

After parsing *R*, we add *s* and *t* as a unique entry and exit node to *G*^*k*^. We say that an (*s*, *t*)-walk $w$ in *Dspells* a word $v$ where $v$ is the concatenation of characters spelled by nodes in $w$. The set of *k*-mers of (*s*, *t*)-walk $w$ is denoted with *K*($w$). Note that due to our assumption that the minimum word length in *L*(*R*) is *k*, this set is not empty. Also, the set of *k*-mers in *D* is defined as the union of all *K*($w$) for all (*s*, *t*)-walks $w$ in *D*. Given the construction and the definitions, the following observations are rather obvious.

Observation 1.

For a text to match a word $w$, it is necessary, but not sufficient, for the text to contain all *k*-mers in $w$. Hence, for a text to match a regex *R*, it has to contain *allk*-mers of at least *one* word in *L*(*R*). Note that the text might not contain a word $w$ even though it contains all *k*-mers since they have to be in the correct order and distance.

Observation 2.

The word spelled by any (*s*, *t*)-walk $w$ in G is in *L*(*R*).

Observation 3.

For any word $v$ ∈ *L*(*R*), there exists an (*s*, *t*)-walk $w$ spelling $v$.

As noted above, the set of *k*-mers in *L*(*R*) is finite, while * and + subexpressions generate infinite repetitive subwords. However, those subwords only contribute a finite number of *k*-mers for filtering. Using this observation, we replaced the * and + subgraphs of *G* with acyclic subgraphs in *G*^*k*^. We then note the following.

**Theorem 4**. For each (*s*, *t*)-walk $w$ in *G*, there exists an (*s*, *t*)-walk $w$′ in *G*^*k*^ with *K*($w$′)⊆*K*($w$).

**Proof**. Of interest are only (*s*, *t*)-walks that traverse a * or + subgraph. Fix any such walk $w$ and assume that it traverses such a subgraph *a* times. If *a* < *k*, then $w$ also exists in *G*^*k*^ and hence $w$′ = $w$ and *K*($w$′) = *K*($w$). If *a* ≥ *k*, then there is in *G*^*k*^ a walk $w$′ that traverses the subgraph *k* − 1 times. There can be more *k*-mers in *K*($w$), but any *k*-mer in *K*($w$′) is also in *K*($w$), since all possible *x*-mers for 0 ≤ *x* ≤ *k* − 1 are generated by *k* − 1 times concatenating the subexpression and using the assumption that no empty words are in * or + subgraphs. Hence, together with the last character before the subexpression and the first character after the expression (if those exist), all possible *k*-mers that a walk can spell are formed.

**Figure 7.**
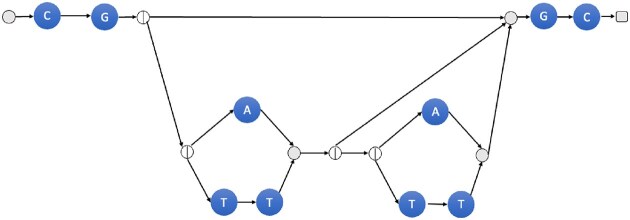
The *k*-graph for *R* = *CG*(*A*|*TT*)*GC and *k* = 3.

The proof is illustrated in the following example in conjunction with Fig. 7.

**Example 2**. Let us assume *R* = *CG*(*A*|*TT*)*GC and *k* = 3. If we construct *G* and then traverse the * expression *a* times, then all possible *k*-mers in (*s*, *t*)-walks are

- *a* = 0: [*CGG*, *GGC*] (one walk);
- *a* = 1: [$CG{\color {red}A}$, ${G\color {red}A}G$, ${\color {red}A}GC$] and [$CG{\color {red}T}$, ${G\color {red}TT}$, ${\color {red}TT}G$, ${\color {red}T}GC$] (two walks);
- *a* = 2: [$CG{\color {red}A}$, ${G\color {red}AA}$, ${\color {red}AA}G$, ${\color {red}A}GC$] and [$CG{\color {red}A}$, ${G\color {red}AT}$, ${\color {red}ATT}$, ${\color {red}TT}G$, ${\color {red}T}GC$] and [$CG{\color {red}T}$, ${G\color {red}TT}$, ${\color {red}TTA}$, ${\color {red}TA}G$, ${\color {red}A}GC$] and [$CG{\color {red}T}$, ${G\color {red}TT}$, ${\color {red}TTT}$, ${\color {red}TTT}$, ${\color {red}TT}G$, ${\color {red}T}GC$] (four walks).

All those walks are also present in *G*^*k*^. Any additional traversal of the * expression might only add some *k*-mers. However, they are not necessary for checking whether *R* has an instance in the text. Important is that we enumerated all *k*-mers in the example. Entering the * expression, we have $CG{\color {red}A}$, $CG{\color {red}T}$, ${G\color {red}AA}$, ${G\color {red}AT}$, and ${G\color {red}TT}$. Exiting the * expression, we have ${\color {red}TA}G$, ${\color {red}AA}G$, ${\color {red}TT}G$, ${\color {red}T}GG$, and ${\color {red}A}GG$.

As mentioned above, this method for bottom-up graph construction can be thought of as a modified version of the Thompson construction. For the *k*-graph *G*^*k*^, the algorithm results in a structure similar to an NFA, but without any cycles. Since *G*^*k*^ is acyclic, we can compute a topological ordering of the nodes, which we can exploit in conjunction with properties of the (H)IBF to determine the positive user bins.

### The collection phase: computing positive bins

In this section, we describe our algorithm for filtering a RegEx *R* by traversing *G*^*k*^ in topological order and maintaining a set of bitvectors that indicates whether the necessary conditions for a set of (*s*, *t*)-walks are met, namely whether all *k*-mers on each walk are present in the same *u* user bins.

**Figure 8.**
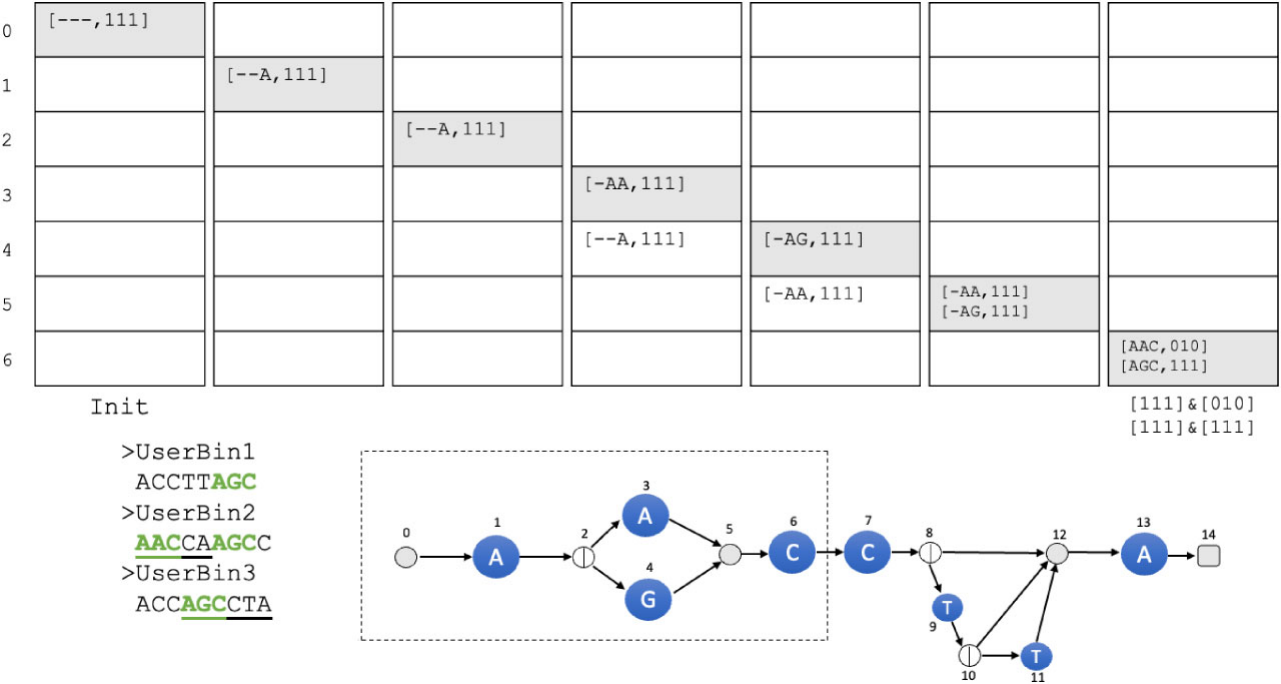
Initialization of the collection phase. We start with [− − −, 111] in *A*[0]. Node 0 points to character node 1. Hence, the entry [− − −, 111] is inserted into *A*[1]. Visiting node 1, there is nothing to absorb, so we leftshift the entry to [− −*A*, 111]. Node 1 points to a split node 2. Hence, the entries are simply moved to *A*[2]. At node 2, there is nothing to absorb and therefore the map entries in *A*[2] are copied to *A*[3] and *A*[4] and deleted in *A*[2]. Next, we visit *A*[3] and leftshift the entry to [− *AA*, 111], since it is a character node spelling *A*. Node 3 points to node 5; therefore, the entries in the map are moved to the map in *A*[5]. We then visit *A*[4] and leftshift the map entry to [− *AG*, 111] since 4 is a character node spelling *G*. It also points to node 5; hence, this entry is inserted in the map of *A*[5]. Node 5 is an exit node; hence, the map entries are moved to *A*[6]. At node 6, there is nothing to absorb and we leftshift the entries to [*AAC*, 111] and [*AGC*, 111], respectively. Since we now have our first full *k*-mer, we update the entries. Since *AAC* is only in the second user bin, the bitvector is updated to 010. *AGC*, on the other hand, is in all three user bins, so the bitvector remains 111.

**Figure 9.**
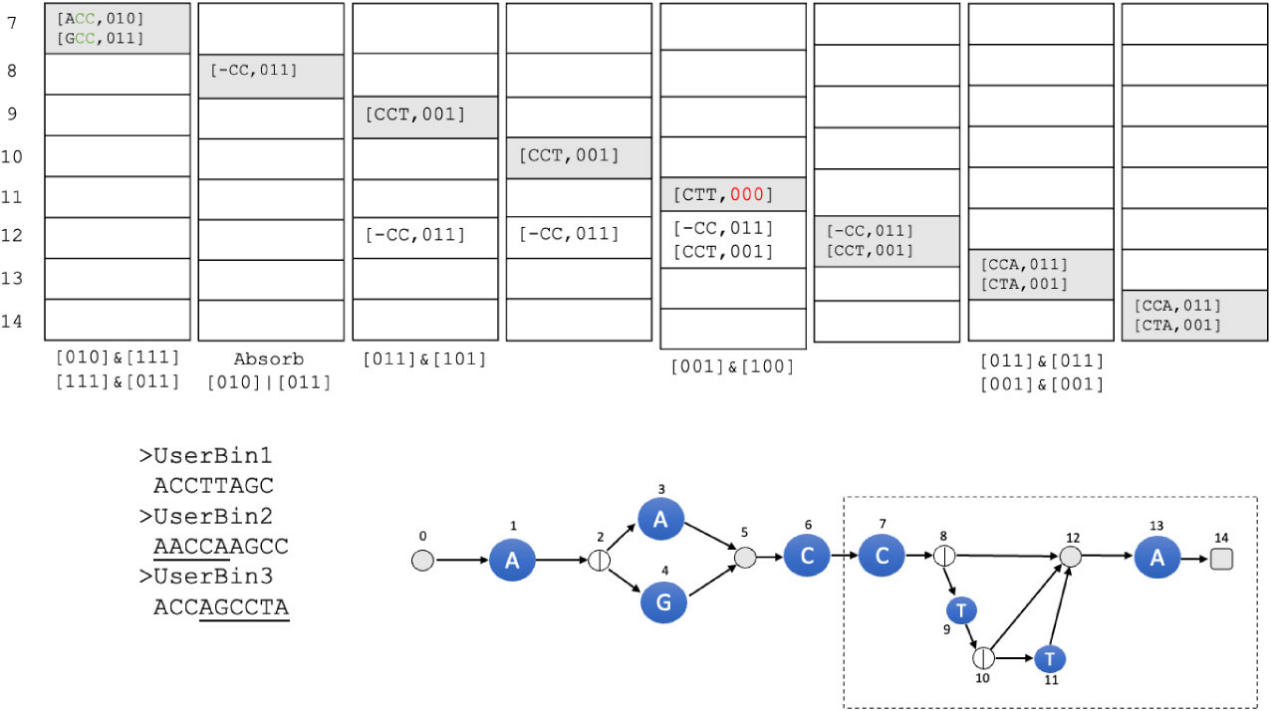
Main part of the collection phase. We continue our traversal of *A* from node 6, which points to the character node 7 spelling *C*. The map entries in 6 are moved, and upon visiting node 7, we see that there is nothing to absorb, so the entries are leftshifted and updated (the update operation is depicted under the respective columns), yielding the entries [*ACC*, 010] and [*GCC*, 011]. They are copied to the map in *A*[8]. Node 8 is a split node and we can absorb the two entries into one. Hence, the list in *A*[8] contains [− *CC*, 011], which is copied to the maps in *A*[9] and *A*[12] and subsequently deleted. Moving to node 9, we leftshift and update the entry, resulting in [*CCT*, 001], and move the entry to *A*[10]. In node 10, there is nothing to absorb and we copy the entry to the maps in *A*[11] and *A*[12]. At node 11, we leftshift the entry and update it to [*CTT*, 000]. Therefore, this entry can safely be deleted. We continue the procedure until node 14, where we now can determine that our RegEx might be contained in user bins 2 and 3.

Figures 8 and 9 show the complete procedure for *R* = *A*(*A*|*G*)CCT*A, *k* = 3, and *u* = 3 user bins.

Assume that for a RegEx *R*, we have constructed *G*^*k*^ with *n* nodes and have determined the topological order of its nodes. We traverse *G*^*k*^ in this order and perform operations depending on the type of node. As a data structure, we maintain an array *A* of size *n* containing hash maps of entries of the form [*s*′↦(*n*, *s*, *b*)], where *n* corresponds to the next node in *G*^*k*^, *s* ∈ (Σ∪{ − })^*k*^ is a *k*-mer, and *b* ∈ {0, 1}^*u*^ is a binning bitvector with a bit for each of the *u* user bins. The key *s*′ is simply the rightmost, *k* − 1 characters of *s*. We start by first setting *A*[0] = [ − ^*k* − 1^↦( − ^*k*^, 1^*u*^)].

We then iterate over *A*, from 0 to *n* − 1 (each index of *A* corresponds to a node in *G*^*k*^) in topological order, and perform operations on the entries in *A* according to the node type. These operations will, for example, query the HIBF for set membership, update the bitvectors in the entry, or condense information between two items that occupy the same index of *A*.

We define the following operations ($\&$ and | are here the bitwise *AND* and *OR* operators):

- **pus** **h** **([n,s,b])**: Inserts a new entry into *A* by checking where the outgoing arc of *n* points to. **push** happens every iteration regardless of the type of node being processed. Each time a **push** is performed, the entry being pushed is removed from its previous location in *A*.
- **[*n*_*r*_, *s*_*r*_, *b*_*r*_]**=**absorb** **([*n*_1_, *s*_1_, *b*_1_], [*n*_2_, *s*_2_, *b*_2_])**: with *s*_*r*_, *s*_1_, *s*_2_ ∈ Σ^*k*^ and *b*_*r*_, *b*_1_, *b*_2_ ∈ {0, 1}^*u*^. If a **push** results in a hash collision, then the bitvectors of the two colliding entries are OR’ed and a single entry is kept that maintains the resulting bitvector, i.e. [*n*_*r*_, *s*_*r*_, *b*_*r*_], where *n*_*r*_ = *n*_1_=*n*_2_ and *b*_*r*_ = *b*_1_|*b*_2_, and *s*_*r*_ = −*c*_2_…*c*_*k*_, where *s*_1_ = *c*_1_…*c*_*k*_. For example, **[*n*_*i*_, −*CC*, 011]= absorb([*n*_*i*_, *ACC*, 001], [*n*_*i*_, *GCC*, 010])**.
- **leftshift** **(*e*_1_ = [*n*_1_, *s*_1_, *b*_1_], *c*)**: with *c* ∈ Σ. This operation replaces in the entry *e*_1_ the *k*-mer *s*_1_ = *c*_1_…*c*_*k*_ with the *k*-mer *s*_2_ = *c*_2_…*c*_*k*_*c*. For example, leftshift([*n*_*i*_, *CAG*, 010], *A*) will change the entry to [*n*_*i*_, *AGA*, 010].
- **update** **(*e* = [*n*, *s*, *b*])**: After querying the HIBF with a *k*-mer *s*, we obtain the bitvector *r*. We then change in *e* the bitvector *b* to the result of $b\, \& \, r$. For example, update([*n*, *ACG*, 011]) might return *r* = 010 and hence will change the entry to [*n*, *ACG*, 010]. If *b* is 0^*u*^ after the update, we consider this walk a dead end and delete the entry from the map without a **push**.

We start our traversal at node 0 with the entry [− ^*k*^, 1^*u*^] in the map *A*[0] and copy the entries of the hashmap to *A*[1]. Assume we are at node *i* > 0 in the graph.

- If *i* is a character node, iterate over entries *e*_1_, …, *e*_*x*_ and call **leftshift**(*e*, *c*). If the *k*-mer of an entry *e* is complete (i.e. after the initialization phase), call **update**(*e*). Finally, **push** all the results into the hash map at *A*[*j*] corresponding to incident node *j*.
- If *i* is a split node, iterate over entries *e*_1_, …, *e*_*x*_. Then **push** all hashmap entries to the hash maps of all incident nodes at *A*[*j* > *i*] and delete them in the map of *i*.
- If *i* < *n* − 1 is an exit node, **push** all hash map entries to the map of node *i* + 1.
- If *i* = *n* − 1, then the node is the last exit node, and we report the user bins in the hash map.

By utilizing these few operations during traversal, the TetRex algorithm is able to seamlessly interface with the (H)IBF and maintain information about which bins contain the necessary *k*-mers for a match in the bitvectors. The choice to traverse the NFA in topological order allows us to exploit fundamental, logical bit operations that act as an elegant reflection of RegExes that contains concatenations and disjunctions. Traversing in topological order and pruning thereby also allows us to avoid overflow of a stack/queue that might occur by traversal with a typical depth-first or breadth-first search.

In comparison with the gram-driven NFA proposed by Qiu *et al.*, TetRex is able to extract all the necessary *k*-mers directly from the NFA, without having to build an additional, separate structure. This is extremely helpful for RegExes that encode a large number of *k*-mers as TetRex avoids having to explicitly store all of these in memory. All the necessary information is symbolically represented in the bitvectors. Instead, the number of split nodes is what determines the worst-case scenario, and this is often ameliorated by pruning dead end walks or by absorbing entries into one another.

### Algorithmic analysis

**Figure 10.**
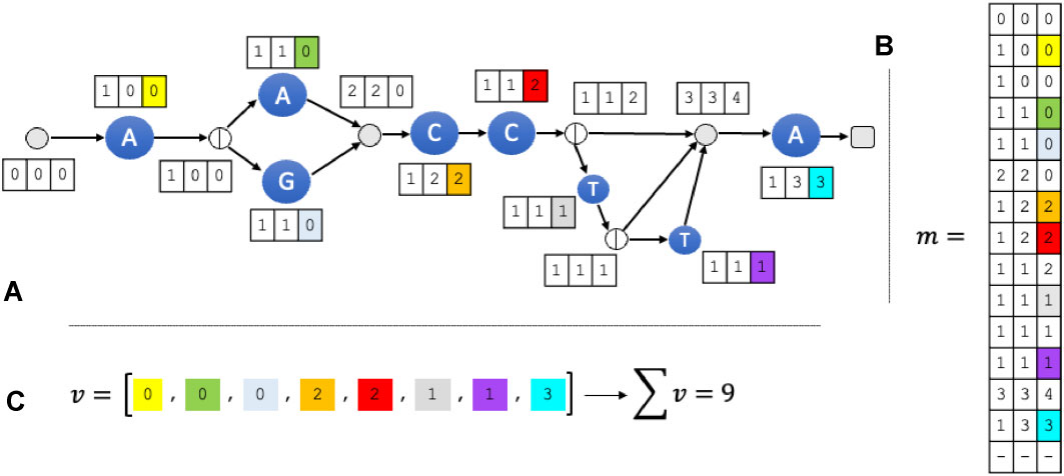
Illustration of how to compute the *k*-mer complexity of the RegEx *R* = *A*(*A*|*G*)CCT*A. (**A**) The *k*-graph of *R* annotated with the final count vectors. (**B**) The corresponding matrix *m*. (**C**) The vector $v$ of *k*-mer counts and the overall complexity.

Here, we describe the algorithmic complexity of our method for collecting *k*-mers from *G*^*k*^. Because the collection phase makes use of *k*-mers, and not single characters, we define the time complexity to be *c*_*k*_, where *c*_*k*_ refers to the *k*-mer complexity, i.e. the absolute number of *k*-mers encoded by a RegEx. Computing this can be done by a simple traversal of the graph and updating a vector. In detail, we describe the procedure to compute *c*_*k*_ below and illustrate it in Fig. 10.

To calculate *k*-mer complexity, we first initialize a matrix *m* of zeroes where the *i*th row corresponds to the *i*th node in *G*^*k*^ in topological order and the number of columns is equal to *k*. We also initialize an empty vector $v$ that has *c* entries, where *c* is the number of character nodes (see Fig. 10C), to store *k*-mer counts. We then iterate over the nodes from *G*^*k*^ in topological order and change *m* according to two procedures. Let *i* = 0 be an index to fill $v$.

- *Character*: If the node corresponds to a character node:
  - Shift the contents of the corresponding row in *m* to the right by one column.
  - Set the first column value to 1.
  - Write the last value of that row into $v$[*i*] and increment *i*.
  - Perform default procedure.
- *Default*: Add the contents of that row to all the nodes downstream of the current node.

The algorithm terminates at the match node and sums the contents of $v$, which is the *k*-mer complexity *c*_*k*_ of the RegEx. The practical relationship between *k*-mer complexity and experimental runtime is explored further in the ‘Discussion’ section.

### Workflow summary

In summary, the database is divided into *u* parts, which we call *user bins* (there can be hundreds of thousands) and are indexed using an (H)IBF. Then, for a given regex *R*, we construct a graph *G*^*k*^ encoding all words in the language represented by *R*.

We then traverse *G*^*k*^ and use the HIBF to compute all user bins that might contain an instance of *R* by maintaining a set of possible accepting paths. Finally, we scan all user bins that might contain an instance with a standard linear search algorithm for *R*.

## Results

In this section, we evaluate the implementation of our algorithm using the HIBF index and compare it to other approaches. Most approaches mentioned earlier (e.g. Treematch, FREE, BEST, and LPMS) are not available to test. The only available filtering approach for regexes we could find is the now discontinued Google Code Search engine, denoted csearch. Additionally, we also include the standard UNIX command egrep (identical to grep -E) as a baseline for both nucleotide and protein searches. We also benchmark the tool ripgrep, a command-line tool written in Rust, based on the RE2 regex engine, that implements numerous optimizations for searching both large files and directories. For Prosite patterns, we also test the Prosite scan tool that is provided with Prosite. All experiments were conducted in the same local macOS Sonoma 14.5, 64-bit environment using 16 GB of RAM and an Apple M1 chip. We conducted two experiments: one artificial using the nucleotide alphabet and one using the amino acid alphabet and the SwissProt database [1]. The data and experiments were prepared as follows.

### Experimental set-up

- Amino acids: The Prosite database describes protein domains, families, and functional sites. It also provides the patterns and profiles to identify them [1]. Prosite represents patterns in an extended RegEx syntax. SwissProt is the high-quality, manually annotated, and non-redundant portion of the UniProt protein reference sequence database. We extracted 116 regexes from PROSITE (see Supplementary Table S2 for a list) and searched for them in the SwissProt database. We divided SwissProt into 1024 user bins (FASTA files). We compare against csearch, egrep, and ripgrep, as well as the Prosite’s search tool, Prosite scan, which we regard as the gold standard. We used *k* = 6 for TetRex with an index size of 186 MiB. See Supplementary Fig. S2.1 for a list of the commands used to perform this experiment.
- Nucleotides: Using mason [14], we constructed a random DNA text of 4 GB that was divided into 8192 = 2^13^ user bins (files), each containing about half a million (2^19^) characters. We constructed five different regexes (see Supplementary Fig. S1) and inserted, for four of these expressions, two random instances in a random user bin, such that one instance spans only one line and the other two lines. Hence, 10 accepted words (constructed randomly) were in five randomly selected files. Also, to highlight what could be a worst-case scenario for many tools, we ensured that for RegEx number 5, no hits were present in the text. We compare TetRex against csearch, egrep, and ripgrep and use *k* = 13 for TetRex with an index size of 5.4 GiB.

Note that we run a modified version of csearch that does not include the time to load the index in the final, reported runtime. Additionally, TetRex was also modified from its default behaviour and does not compute and check the reverse strand during DNA search to make it comparable to the others. Finally, all tools were allocated a single thread, which results in significant performance differences for ripgrep.

### Benchmarking comparison

**Figure 11.**
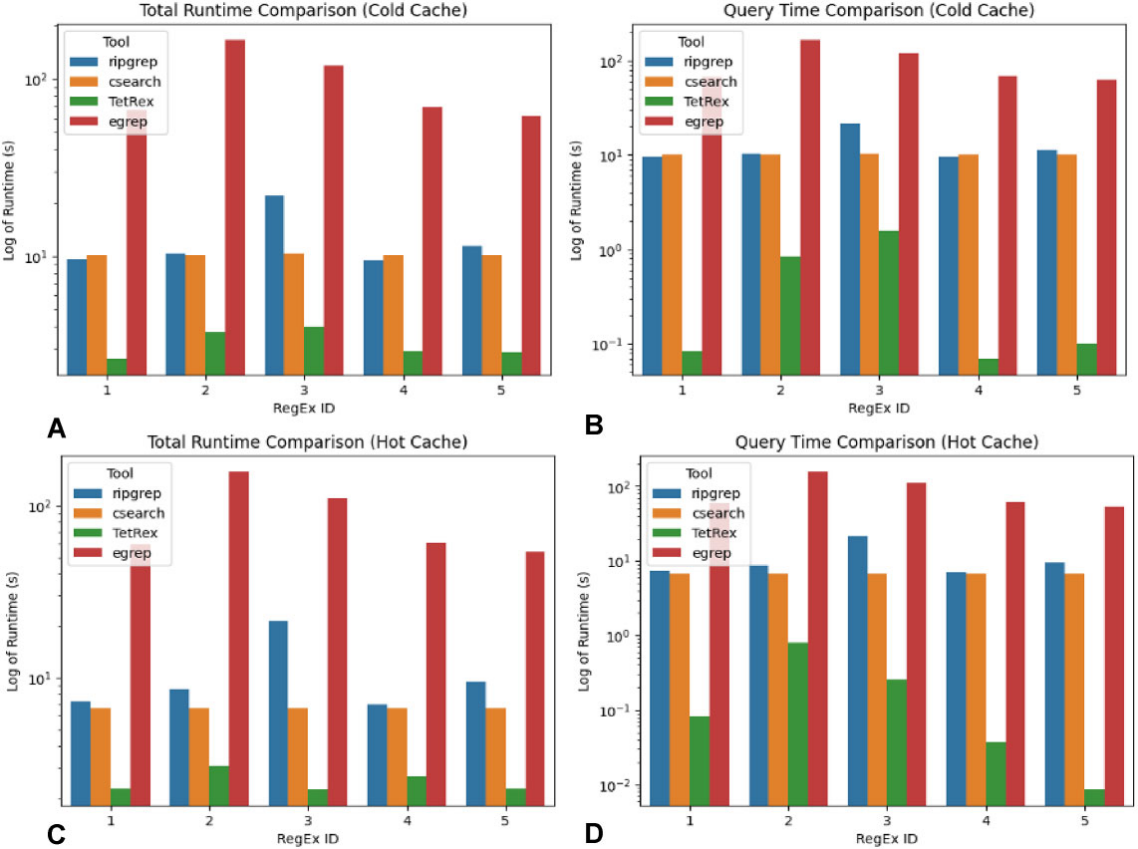
Runtime comparison plotted in logarithmic scale. (**A**) Total runtime comparison after cache clearing and including the time to load the HIBF index into memory. (**B**) Runtime comparison of only query times after clearing the cache. (**C**) Total runtime comparison after having previously read the index into memory. (**D**) Runtime comparison of only query times after having previously loaded the index.

In Fig. 11, we display the results of the nucleotide queries and report the raw runtimes in Supplementary Table S1. As expected, we observe that the trigram filtering employed by csearch is not sufficient for filtering out DNA queries. The index is unable to rule out any bins and the final search runtime is very similar to that of a linear search (egrep). Additionally, we find that csearch is unable to match over linebreaks and thus does not report all occurrences of a RegEx. For egrep, we were able to solve this problem by preprocessing the database so that all fasta sequences span only one line, but this solution did not work for csearch. On average, we find the TetRex query time to be 374 times faster than egrep, 45 times faster than ripgrep, and 28 times faster than csearch. The TetRex filtering approach is notably able to restrict the search space to only the bins where a positive hit exists. We also highlight the advantage in the TetRex approach when no match is present.

**Figure 12.**
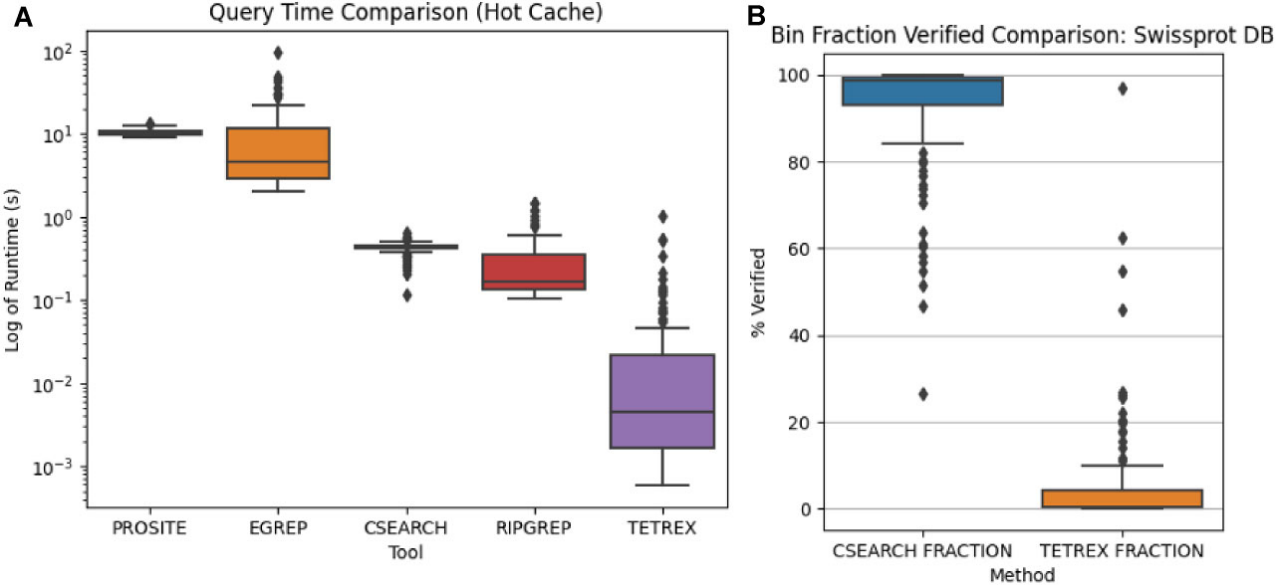
Performance of tools searching 116 prosite patterns over the SwissProt database. (**A**) Average runtime plotted in logarithmic scale. (**B**) Percentage of bins requiring a linear scan for both csearch and TetRex.

In Fig. 12, we display the results of the amino acid queries. Notably, while both csearch and TetRex vastly outperform both prosite and egrep, the csearch behaviour is slightly unexpected. While one might expect that a trigram index would be suitable for the larger alphabet size of amino acids, the csearch trigram filter is surprisingly often unable to narrow the search space to any meaningful extent. Instead, it frequently performs a linear search over the entire database. However, with the exception of some particularly pathological RegEx, the filtering scheme employed by TetRex is often able to narrow down the search space by nearly 100%, therefore only needing to verify a small number of bins (see also Supplementary Table S3). We find that, on average, TetRex is 246, 239, 10, and 7 times faster than prosite, egrep, csearch, and ripgrep, respectively.

## Discussion

### Utility

Given TetRex’s reliance on *k*-mers, it is important to demonstrate the viability of this approach. Namely, are biological motifs even long enough to be broken down into *k*-mers?

**Figure 13.**
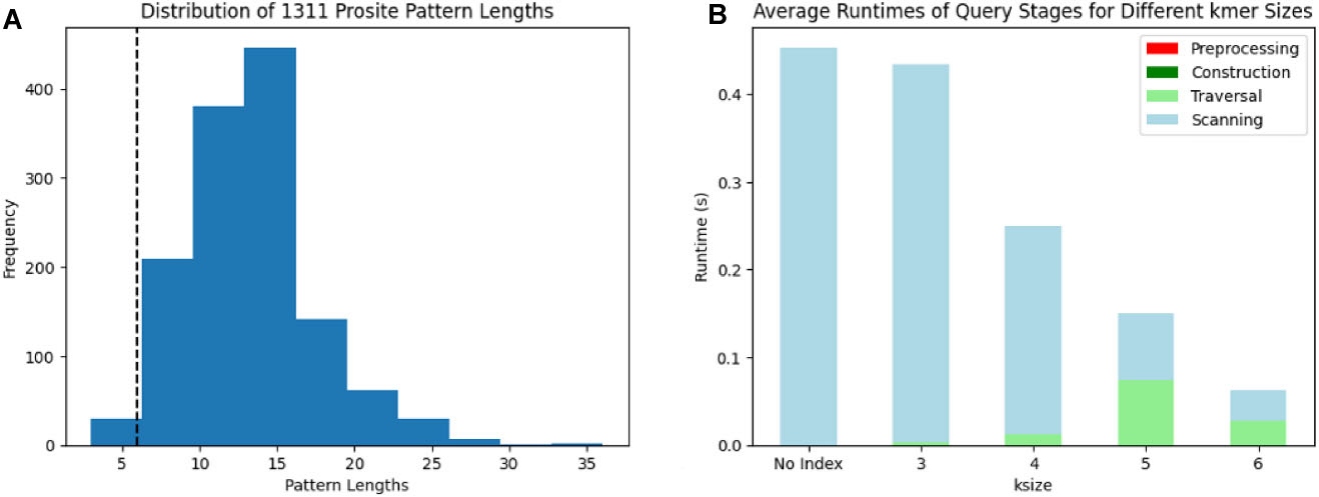
(**A**) Distribution of a lower-bound estimate for the length of all 1311 patterns in the Prosite DB. A vertical mark is shown at 6 (the *k*-mer length used for benchmarking). (**B**) The average runtime of the different stages of TetRex over the 116 Prosite patterns used in benchmarking for a variety of values of *k*. The leftmost bar makes no use of the index and therefore the entire runtime is devoted to linear scanning.

Figure 13A depicts a lower-bound estimate of the distribution of Prosite pattern lengths with a vertical boundary at 6. The methodology for computing pattern length is biased towards shorter estimations because it merely counts the number of concatenation operators ‘-’ in a pattern and adds one. In fact, many patterns will contain min-max quantifiers and actually will describe much longer patterns. Supplementary Table S4 contains the patterns found to be <6 characters in length.

Using the described methodology for estimating pattern length, only 15 patterns would be unsuitable for search with *k*= 6. However, as mentioned above, patterns 5, 6, 10, and 15 actually contain quantifiers that describe longer patterns, bringing the final count of unsuitable patterns down to 11 out of 1311 (<1%).

### Query stage runtime analysis

The TetRex query subcommand is composed of several stages:

- Preprocessing: validating input queries, shunting-yard algorithm.
- Graph construction: constructing the *k*-graph.
- Graph traversal: the *k*-mer collection step.
- Linear scanning: file I/O and RegEx matching.

Figure 13B consistently indicates that, for an increasing length of *k*, the overall runtime decreases. It also clearly indicates that both the preprocessing and graph construction stages have negligible runtimes compared to the graph traversal and scanning stages. This is consistent with our analysis of the algorithm for constructing *G*^*k*^, as these RegExes contain no repeat operators, nested or otherwise.

### *k*-mer complexity and runtime

Interpreting the time for the traversal stage is slightly more difficult. As the *k* grows, the number of possible *k*-mers generally grows as well. RegExes that contain many contiguous, multi-character disjunctions can therefore produce an enormous number of *k*-mers.

**Figure 14.**
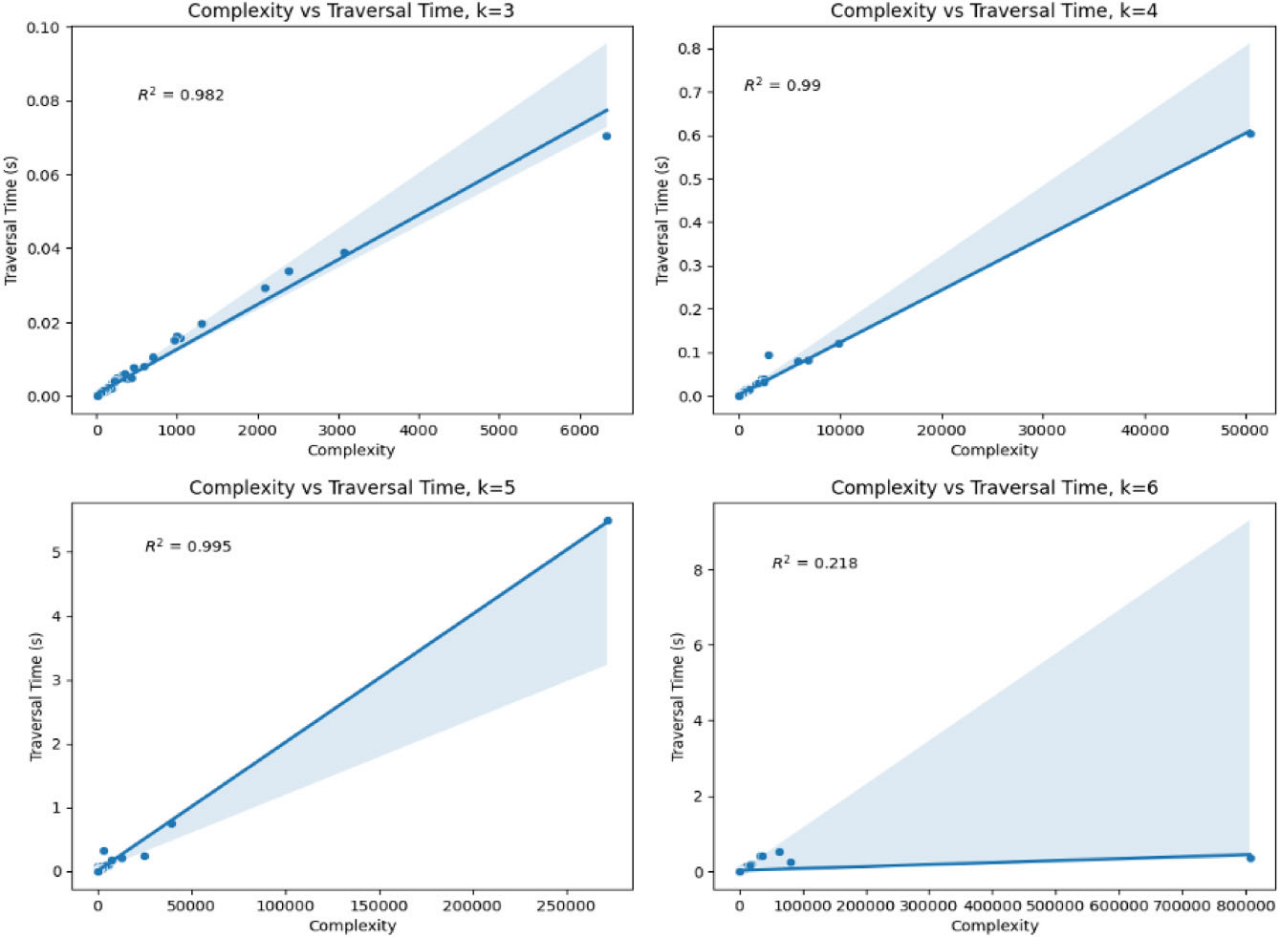
Correlation of *k*-mer complexity with traversal time for different values of *k*. The (light blue) shaded region shows a 95% confidence interval of the linear model’s prediction. The *R*^2^ values provided suggest a strong correlation except for when *k* = 6.

Figure 14 shows that *k*-mer complexity is often a strong predictor of the traversal runtime. However, there is a clear discrepancy between *k* = 5 and 6. In fact, it is a single RegEx PS00141 that causes this discrepancy. When *k* = 5, PS00141 has a complexity of 271 696 and a traversal time of 5 s. When *k*= 6, it has a complexity of 806 208 and a traversal time of 0.354 s. Despite the higher complexity, when the *k*-mer size is larger, the traversal time is much shorter. We suspect that the larger *k*-mer size allows the collection step to prune dead end paths much sooner in the traversal.

**Figure 15.**
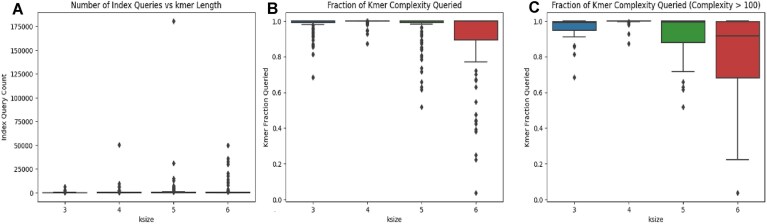
(**A**) The raw number of queries made to the index for different *k*-mer sizes. (**B**) The fraction of *k*-mers being queried for a regex compared to that RegExes *k*-mer complexity. (**C**) The fraction of *k*-mers that are queried for RegEx’s with a *k*-mer complexity >100. Note that some *k*-mers will not be queried since the walk they appear in might be pruned before the respective *k*-mer is ever reached.

This hypothesis is supported by Fig. 15B, which indicates that for *k* < 6, almost all *k*-mers end up being queried. However, for *k* = 6 it is slightly more common that a smaller fraction is queried. Additionally, in Fig. 15C, we show that for higher complexity queries, *k* = 6 often shows an even smaller fraction of *k*-mers being queried.

In practice, while TetRex employs a number of strategies to avoid long traversal times, this is ultimately determined by both the content of the database, i.e. pruning dead end paths, and properties of the RegEx itself, i.e. *k*-mer complexity and absorption, and may be very difficult to predict. We note, however, that the vector $v$ described in the computation of *k*-mer complexity allows for further opportunities to avoid the worst-case scenario by giving insight into what portions of the graph generate a large amount of *k*-mer complexity. These can subsequently be excluded from the collection algorithm or even replaced by a min-max quantifier.

## Conclusion

We presented a new filtering approach for searching regexes in large databases with applications for nucleotide and amino acid searches. We achieve this by dividing the database into *user bins* and using an HIBF [10] to index the text in the bins for *k*-mer queries. We convert a regex into an acyclic graph that we traverse. During traversal, we query the HIBF, resulting in only a few of the user bins being searched as opposed to the entire text.

We implemented our approach and evaluated it on two bioinformatics challenges, one for amino acids and one for nucleotides. In both cases, we can significantly reduce the search space, resulting in search times of mere fractions of a second, which outperforms other state-of-the-art tools by orders of magnitude.

We note that despite the already sizable performance improvements provided by the TetRex approach, numerous improvements to both performance and functionality still remain possible: Recent algorithmic advances to seeding approaches (i.e. minimizers [15] and strobemers [16]) will allow for smaller index sizes, more intelligent *k*-graph construction will allow for more effective narrowing of the search space, and multithreading will allow for reduction in verification time. Practically, we could also allow for queries in a reduced alphabet space [17,18]. This is more amenable for comparison-based protein search (used in tools like Diamond [19] or Lambda3 [20]).

The TetRex approach can also be extended to more powerful queries. Specifically, those containing min-max quantifiers. For example, if two small substrings are 11 or 12 characters apart and there are no or very few restrictions on the characters between the substrings, it will be advantageous not to use all the *k*-mers between the substrings but instead test user bins for the two substrings being present at a distance of 11 or 12. This happens, for example, in the Prosite pattern PDOC00284 for 11-S plant seed storage proteins: N-G-x-[DE](2)-x-[LIVMF]-C-[ST]-x(11,12)-[PAG]-D. Furthermore, some biological patterns also make use of anchors like $ and ^ to describe patterns that must occur at the N or C terminus of a peptide. To address these, we plan to implement a method for encoding *k*-mers that occur at the beginning or end of a FASTA record.

Additionally, since RegExes also find use in numerous other fields beyond bioinformatics, the TetRex algorithm could, of course, be extended to search code and other forms of text. While the indexing, graph construction, traversal, and scanning workflow could mostly remain the same, the optimized parsing of FASTA file formats, encoding of biological molecules, and extra work devoted to scanning both forward and reverse strands of DNA would have to be modified. It is likely that a bioinformatics-specific tool would be provided separately from a more general-use tool.

In conclusion, we believe that TetRex, and its underlying algorithms, provide valuable contributions to speed up the search for regexes. While RegEx has applications to many domains, we think it will be particularly useful in bioinformatics. The code is freely available at https://github.com/remyschwab/TetRex.

## Supplementary Material

lqaf039_Supplemental_Files

## Data Availability

The code is freely available at https://github.com/remyschwab/TetRex. The TetRex repository is available at https://doi.org/10.5281/zenodo.15056086. The analysis is available at https://doi.org/10.5281/zenodo.15056051.
