# Supplementary material for: TetRex: a novel algorithm for index-accelerated search of highly conserved motifs
Source: NAR Genom Bioinform. 2025 Apr 17;7(2):lqaf039. doi: 10.1093/nargab/lqaf039 (PMC12004226; doi:10.1093/nargab/lqaf039)
Supplement: lqaf039_Supplemental_Files [file lqaf039_supplemental_files.zip › supplementary_materials_2.pdf]

DNA Simulation Benchmark

```
## Ripgrep
sync && sudo purge
time rg -j 1 -o -U "ATGTTT(A|G)TTTT?
CTTATTA(TTT|CCC)CTTACTCTCACTAGT" small_sim
sync && sudo purge
time rg -j 1 -o -U "
(CT(A|C|G|T)|TT(A|G))ATGGC(A|C|G|T)
(GA(A|G)|CA(G|A))GG(A|C|G|T)
(CT(A|C|G|T)|TT(A|G))TA(T|C)AA(C|T)" small_sim
sync && sudo purge
time rg -j 1 -o
"GC(A|C|G|T)GG(A|C|G|T)GC(A|C|G|T)GC(A|C|G|T)GC(A|C|G
|T)GC(A|C|G|T)GG(A|C|G|T)GC(A|C|G|T)GT(A|C|G|T)GT(A|C
|G|T)GG(A|C|G|T)GG(A|C|G|T)
(CT(A|C|G|T)|TT(A|G))GG(A|C|G|T)GG(A|C|G|T)TA(T|C)"
linearized_sim ## Had to use the linearized version
for some reason
sync && sudo purge
time rg -j 1 -o -U "ACACACGGTTTTGGAG*AC" small_sim
sync && sudo purge
time rg -j 1 -o -U
"TTAACGTGATGATCAGCTACGCATAAATAGATA" small_sim
## TetRex
tetrex index -k 13 -m na -o sim_genomes
small_sim/*.fasta
sync && sudo purge
time tetrex query sim_genomes.ibf "ATGTTT(A|G)TTTT?
```

```
CTTATTA(TTT|CCC)CTTACTCTCACTAGT"
sync && sudo purge
time tetrex query sim_genomes.ibf "
(CT(A|C|G|T)|TT(A|G))ATGGC(A|C|G|T)
(GA(A|G)|CA(G|A))GG(A|C|G|T)
(CT(A|C|G|T)|TT(A|G))TA(T|C)AA(C|T)"
sync && sudo purge
time tetrex query sim_genomes.ibf
"GC(A|C|G|T)GG(A|C|G|T)GC(A|C|G|T)GC(A|C|G|T)GC(A|C|G
|T)GC(A|C|G|T)GG(A|C|G|T)GC(A|C|G|T)GT(A|C|G|T)GT(A|C
|G|T)GG(A|C|G|T)GG(A|C|G|T)
(CT(A|C|G|T)|TT(A|G))GG(A|C|G|T)GG(A|C|G|T)TA(T|C)"
sync && sudo purge
time tetrex query sim_genomes.ibf
"ACACACGGTTTTGGAG*AC"
sync && sudo purge
time tetrex query sim_genomes.ibf
"TTAACGTGATGATCAGCTACGCATAAATAGATA"
## csearch
./cindex $HOME/repos/TetRex/data/small_sim
sync && sudo purge
csearch -verbose "ATGTTT(A|G)TTTT?
CTTATTA(TTT|CCC)CTTACTCTCACTAGT"
sync && sudo purge
csearch -verbose "(CT(A|C|G|T)|TT(A|G))ATGGC(A|C|G|T)
(GA(A|G)|CA(G|A))GG(A|C|G|T)
(CT(A|C|G|T)|TT(A|G))TA(T|C)AA(C|T)"
sync && sudo purge
csearch -verbose
"GC(A|C|G|T)GG(A|C|G|T)GC(A|C|G|T)GC(A|C|G|T)GC(A|C|G
|T)GC(A|C|G|T)GG(A|C|G|T)GC(A|C|G|T)GT(A|C|G|T)GT(A|C
|G|T)GG(A|C|G|T)GG(A|C|G|T)
```

```
(CT(A|C|G|T)|TT(A|G))GG(A|C|G|T)GG(A|C|G|T)TA(T|C)"
sync && sudo purge
csearch -verbose "ACACACGGTTTTGGAG*AC"
sync && sudo purge
csearch -verbose "TTAACGTGATGATCAGCTACGCATAAATAGATA"
## egrep
sync && sudo purge
time egrep -ro "ATGTTT(A|G)TTTT?
CTTATTA(TTT|CCC)CTTACTCTCACTAGT" linearized_sim
sync && sudo purge
time egrep -ro "(CT(A|C|G|T)|TT(A|G))ATGGC(A|C|G|T)
(GA(A|G)|CA(G|A))GG(A|C|G|T)
(CT(A|C|G|T)|TT(A|G))TA(T|C)AA(C|T)" linearized_sim
sync && sudo purge
time egrep -ro
"GC(A|C|G|T)GG(A|C|G|T)GC(A|C|G|T)GC(A|C|G|T)GC(A|C|G
|T)GC(A|C|G|T)GG(A|C|G|T)GC(A|C|G|T)GT(A|C|G|T)GT(A|C
|G|T)GG(A|C|G|T)GG(A|C|G|T)
(CT(A|C|G|T)|TT(A|G))GG(A|C|G|T)GG(A|C|G|T)TA(T|C)"
linearized_sim
sync && sudo purge
time egrep -ro "ACACACGGTTTTGGAG*AC" linearized_sim
sync && sudo purge
time egrep -ro "TTAACGTGATGATCAGCTACGCATAAATAGATA"
linearized_sim
```

Supplementary Figure 2.1: The list of commands performed for the simulated DNA evaluation. These can be used to reproduce the results of the experiment.
