# Supplementary material for: TetRex: a novel algorithm for index-accelerated search of highly conserved motifs
Source: NAR Genom Bioinform. 2025 Apr 17;7(2):lqaf039. doi: 10.1093/nargab/lqaf039 (PMC12004226; doi:10.1093/nargab/lqaf039)
Supplement: lqaf039_Supplemental_Files [file lqaf039_supplemental_files.zip › supp_info_revised.pdf]

Supplementary Materials

a. ATGTTT(A|G)TTTT?CTTATTA(TTT|CCC)CTTACTCTCACTAGT
b. (CT(A|C|G|T)|TT(A|G))ATGGC(A|C|G|T)(GA(A|G)|CA(G|A))GG(A|C|G|T)(CT(A|C|G|T)|TT(A|G))TA(T|C)AA(C|T)
c. GC(A|C|G|T)GG(A|C|G|T)GC(A|C|G|T)GC(A|C|G|T)GC(A|C|G|T)GC(A|C|G|T)GG(A|C|G|T)GC(A|C|G|T)GT(A|C|G|T)GT(A|C|G|T)GG(A|C|G|T)GG(A|C|G|T)(CT(A|C|G|T)|TT(A|G))GG(A|C|G|T)GG(A|C|G|T)TA(T|C)
d. ACACACGGTTTTGGAG*AC
e. TTAACGTGATGATCAGCTACGCATAAATAGATA

Supplementary Figure 1: Regular Expressions used for DNA simulation benchmark

RegEx ID	Tool	Cache	total time (s)	query time (s)
1	ripgrep	cold	9.562	9.562
2	ripgrep	cold	10.341	10.341
3	ripgrep	cold	21.840	21.840
4	ripgrep	cold	9.542	9.542
5	ripgrep	cold	11.385	11.385
1	ripgrep	hot	7.275	7.275
2	ripgrep	hot	8.595	8.595
3	ripgrep	hot	21.271	21.271
4	ripgrep	hot	7.019	7.019
5	ripgrep	hot	9.465	9.465
1	csearch	cold	10.100	10.100
2	csearch	cold	10.027881	10.027881
3	csearch	cold	10.301633	10.301633
4	csearch	cold	10.095272	10.095272
5	csearch	cold	10.091027	10.091027
1	csearch	hot	6.697	6.697
2	csearch	hot	6.641756	6.641756
3	csearch	hot	6.635184	6.635184
4	csearch	hot	6.640707	6.640707
5	csearch	hot	6.637957	6.637957
1	TetRex	cold	2.627	0.084234
2	TetRex	cold	3.732	0.847021
3	TetRex	cold	4.015	1.57228
4	TetRex	cold	2.887	0.069435
5	TetRex	cold	2.849	0.100136
1	TetRex	hot	2.271	0.081805
2	TetRex	hot	3.061	0.79885
3	TetRex	hot	2.244	0.256552
4	TetRex	hot	2.691	0.03654
5	TetRex	hot	2.273	0.008594
1	egrep	cold	66.68	66.68
2	egrep	cold	166.76	166.76
3	egrep	cold	119.85	119.85
4	egrep	cold	69.73	69.73
5	egrep	cold	62.23	62.23
1	egrep	hot	59.661	59.661
2	egrep	hot	157.59	157.59
3	egrep	hot	110.53	110.53
4	egrep	hot	60.97	60.97
5	egrep	hot	53.676	53.676

Supplementary Table 1: Raw timings and cache status for RegEx DNA benchmarks

No.	ID	PATTERN
1	PS00032	[LIVMFE]-[FY]-P-W-M-[KRQTA]
2	PS00033	L-M-A-[EQ]-G-L-Y-N
3	PS00058	G-F-R-G-E-[AG]-L
4	PS00064	[LIVMA]-G-[EQ]-H-G-[DN]-[ST]
5	PS00069	D-H-[YF]-L-G-K-[EQK]
6	PS00092	[LIVMAC]-[LIVFYWA]-{DYP}-[DN]-P-P-[FYW]
7	PS00093	[LIVMF]-T-S-P-P-[FY]
8	PS00115	Y-[ST]-P-[ST]-S-P-[STANK]
9	PS00116	[YA]-[GLIVMSTAC]-D-T-D-[SG]-[LIVMFTC]-{LA}-[LIVMSTAC]
10	PS00120	[LIV]-{KG}-[LIVFY]-[LIVMST]-G-[HYWV]-S-{YAG}-G-[GSTAC]
11	PS00125	[LIVMN]-[KR]-G-N-H-E
12	PS00134	[LIVM]-[ST]-A-[STAG]-H-C
13	PS00141	[LIVMFGAC]-[LIVMTADN]-[LIVFSA]-D-[ST]-G-[STAV]-[STAPDENQ]-{GQ}-[LIVMFSTNC]-{EGK}-[LIVMFGTA]
14	PS00142	[GSTALIVN]-{PCHR}-{KND}-H-E-[LIVMFYW]-{DEHRKP}-H-{EKPC}-[LIVMFYWGSPQ]
15	PS00154	D-K-T-G-T-[LIVM]-[TI]
16	PS00161	K-[KR]-C-G-H-[LMQR]
17	PS00171	[AVG]-[YLV]-E-P-[LIVMEPKST]-[WYEAS]-[SAL]-[IV]-[GN]-[TEKDVS]-[GKNAD]
18	PS00177	[LIVMA]-{R}-E-G-[DN]-S-A-{F}-[STAG]
19	PS00191	[FY]-[LIVMK]-{I}-{Q}-H-P-[GA]-G
20	PS00221	[HNQA]-{D}-N-P-[STA]-[LIVMF]-[ST]-[LIVMF]-[GSTAFY]
21	PS00224	F-L-A-[QH]-[QE]-E-S
22	PS00226	[IV]-{K}-[TACI]-Y-[RKH]-{E}-[LM]-L-[DE]
23	PS00227	[SAG]-G-G-T-G-[SA]-G
24	PS00231	C-[DE]-[YF]-N-R-D
25	PS00267	F-[IVFY]-G-[LM]-M-[G>]
26	PS00290	[FY]-{L}-C-{PGAD}-[VA]-{LC}-H
27	PS00291	A-G-A-A-A-A-G-A-V-V-G-G-L-G-G-Y
28	PS00307	[LIV]-[STAG]-V-[DEQV]-[FLI]-D-[ST]
29	PS00308	[LIV]-{LA}-[EDQ]-[FYWKR]-V-{VF}-[LIVF]-G-[LF]-[ST]
30	PS00319	G-[VT]-[EK]-[FY]-V-C-C-P
31	PS00320	G-Y-E-N-P-T-Y-[KRS]
32	PS00321	A-L-[KR]-[IF]-[FY]-[STA]-[STAD]-[LIVMQ]-R
33	PS00322	K-A-P-R-K-[QH]-[LI]
34	PS00336	[FY]-E-[LIVM]-G-S-[LIVMG]-[SA]-K
35	PS00341	I-P-C-C-P-V
36	PS00345	L-[FYW]-[QEDH]-F-[LI]-[LVQK]-{N}-[LI]-L
37	PS00348	M-C-N-S-S-C-[MV]-G-G-M-N-R-R
38	PS00349	R-K-R-K-Y-F-K-K-H-E-K-R
39	PS00355	[RQ]-R-S-A-[RS]-L-S-A-[RKM]-[PL]
40	PS00369	G-[LIVM]-H-[STAV]-R-[PAS]-[GSTA]-[STAMVN]
41	PS00387	D-[SGDN]-D-[PE]-[LIVMF]-D-[LIVMGAC]

42	PS00405	G-Q-D-Q-T-K-Q-Q-I
43	PS00410	L-P-[RKT]-[GD]-[STNKEA]-[GND]-[LIVMG]-[VICA]-T-R
44	PS00415	L-R-R-R-L-S-D-S
45	PS00416	G-H-A-H-[SA]-G-M-G-K-[IV]-K
46	PS00419	C-D-G-P-[GE]-R-G-G-T-C
47	PS00431	G-[EQ]-T-V-V-P-G-G-T
48	PS00449	[STAGN]-{E}-[STAG]-[LIVMF]-R-L-{LP}-[SAGV]-N-[LIVMT]
49	PS00485	[SA]-[LIVM]-[NGS]-[STA]-D-D-P
50	PS00496	Y-[KR]-G-[AS]-[AE]-Y
51	PS00501	[GS]-{PR}-S-M-{RS}-[PS]-[AT]-[LF]
52	PS00505	[FY]-P-S-[AGMS]-C-G-K-T-[NS]
53	PS00517	[DEQ]-[KRQT]-[LMF]-E-[FYW]-[LV]-G-D-[SARHG]
54	PS00539	F-[GSTV]-P-R-L-[G>]
55	PS00541	S-K-R-K-Y-R-K
56	PS00557	S-N-H-G-[AG]-R-Q
57	PS00564	[ASL]-[FY]-S-G-G-[LV]-D-T-[ST]
58	PS00569	V-V-H-F-F-K-N
59	PS00572	[LIVMFSTC]-[LIVFYS]-[LIV]-[LIVMST]-E-N-G-[LIVMFAR]-[CSAGN]
60	PS00575	G-[MV]-A-L-F-C-G-C-G-H
61	PS00593	L-[IV]-A-H-[STACH]-Y-[STV]-[RT]-Y-[LIVM]-G
62	PS00599	T-[LIVMFYW]-[STAG]-K-[SAG]-[LIVMFYWR]-[SAG]-{ENKR}-{TNDR}-[SAG]
63	PS00631	[NS]-[TS]-D-A-E-G-R-[LVMI]
64	PS00656	[LIVMYA]-[LIVA]-[LIVT]-[LIV]-E-P-D-[SAL]-[LI]-[PSAG]
65	PS00658	W-[QKR]-[NSD]-[SA]-[LIV]-R-H
66	PS00687	[LIVMFGA]-E-[LIMSTAC]-[GS]-G-[KNLM]-[SADN]-[TAPFV]
67	PS00694	G-[LIVMFY]-N-[LIVM]-K-Y-R-Y-E
68	PS00697	[EDQH]-{K}-K-{VEDI}-[DN]-G-{GLYN}-R-[GACIVM]
69	PS00699	[LIVMFYH]-[LIVMFST]-H-[AG]-[AGSP]-[LIVMNQA]-[AG]-C
70	PS00718	R-R-T-[IV]-[ATN]-K-Y-R
71	PS00721	[GN]-[LIVMS]-K-G-[GST]-[AG]-[AST]-G-[GAS]-G-[YLHRKF]
72	PS00730	[GSARY]-[LIVMF]-[CT]-[LIVMFY]-D-T-C-H
73	PS00733	[YH]-C-[VI]-[SA]-C-A-I-H
74	PS00763	[LIV]-[AGD]-F-P-[CS]-[NG]-Q-F
75	PS00767	P-[GK]-G-[VI]-G-P-[MFI]-T-[IVA]
76	PS00772	V-[DN]-Y-[EQD]-F-V-[DN]-C
77	PS00796	[RA]-N-L-[LIV]-S-[VG]-[GA]-Y-[KN]-N-[IVA]
78	PS00797	Y-K-[DE]-[SG]-T-L-I-[IML]-Q-L-[LF]-[RHC]-D-N-[LF]-T-[LS]-W-[TANS]-[SAD]
79	PS00822	P-F-D-R-H-D-W
80	PS00823	[KR]-[LIM]-K-[DE]-K-[LIM]-P-G
81	PS00826	G-Q-E-N-G-H-V-[KR]
82	PS00858	[LIVM]-[ST]-[KR]-[LIVMF]-E-[ST]-R-P
83	PS00861	G-W-T-L-N-S-A-G-Y-L-L-G-P
84	PS00867	[LIVMF]-[LIMN]-E-[LIVMCA]-N-[PATLIVM]-[KR]-[LIVMSTAC]
85	PS00887	[SGALC]-[LIMF]-[LIVMF]-T-D-[GA]-R-[LIVMFY]-S-[GA]-[GAV]-[ST]

86	PS00906	[SP]-[IVCLAM]-W-[LIVMFYC]-[LM]-R-[QR]-[AVS]-G-R
87	PS00929	S-Q-[IV]-[STGNH]-D-G-Q-[LIV]-Q-[AIV]-[STA]
88	PS00952	L-E-[SA]-V-A-I-[LM]-P-Q-[LI]
89	PS00967	F-[LIVMF]-F-R-P-R-N
90	PS00984	C-F-W-K-Y-C
91	PS00994	R-[LIVM]-[GSAT]-E-V-[GSAR]-A-R-F-[STAIV]-L-D-[GSA]-[LM]-P-G-K-Q-M-[GSA]-I-D-[GSA]-[DAE]
92	PS00996	[LV]-Y-[IVC]-P-R-K-C-S-[SAT]
93	PS01002	[IFAED]-[GA]-[GASF]-N-[PAK]-S-[GTA]-E-[GDEVCF]-[PAGEQV]-[DEQGAV]
94	PS01024	E-F-D-Y-L-K-S-L-E-I-E-E-K-I-N
95	PS01025	[NH]-[AG]-H-[TAD]-Y-H-I-N-S-I-S-[LIVMN]-[NS]-S-D
96	PS01032	[LIVMFY]-[LIVMFYA]-[GSAC]-[LIVM]-[FYC]-D-G-H-[GAV]
97	PS01041	A-E-[KR]-R-E-H-E-[KR]-E-V
98	PS01046	D-G-[PD]-S-A-[GS]-[LIVMCA]-[TA]-[LIVM]
99	PS01062	S-V-A-G-L-G-G-C-P-Y
100	PS01100	L-I-D-I-G-S-G-P-T-[IV]-Y-Q-[LV]-L-[SA]-A-C
101	PS01127	[ELAS]-[LIVMF]-[NVCKGST]-[SCVA]-[QE]-T-D-[FS]-[VLA]-[SAT]-[KRNLAQS]
102	PS01157	G-S-Y-P-S-G-H-T
103	PS01210	F-E-D-[LV]-I-A-[DE]-[PA]
104	PS01235	[LV]-P-[VI]-[VTPI]-[NQLHT]-[FL]-[ATVS]-[AS]-G-G-[LIV]-[AT]-T-P-[AQS]-D-[AGVS]-[AS]-[LM]
105	PS01236	[GARVS]-[LVI]-[ILAV]-[LIVF]-P-G-G-E-S-[TS]-[STAV]
106	PS01266	[QGF]-[WLCF]-G-D-E-[GA]-K-[GA]
107	PS01295	[IVT]-[LIVMC]-[IVT]-[HS]-D-[SGAV]-[AV]-R
108	PS01302	H-N-H-P-[SQ]-G
109	PS01325	P-H-H-D-[SA]-S-T-F
110	PS01336	[SA]-[FY]-[LIV]-L-[STN]-E-S-S-[LIVMF]-F-[LIV]
111	PS01337	[LT]-L-E-[FY]-[AVC]-[DE]-[DE]-[KNQHT]-[LMT]
112	PS01345	C-S-R-C-C-[DE]-[KR]-K-S-C
113	PS01349	[RHQ]-[ST]-W-[GSA]-G-A-R-P-E
114	PS60002	E-G-G-E-L-G-Y
115	PS60009	C-[GA]-E-[ST]-C-[FTV]-[GLTI]-G-[TSK]-C
116	PS60016	C-[IT]-P-S-G-Q-P-C

Supplementary Table 2: Prosite IDs and patterns used for amino acid benchmarking

No.	Real Bin Count	csearch bin count	TetRex bin count
1	7	479	561
2	3	991	3
3	7	991	8
4	3	993	10
5	1	959	4
6	43	961	640
7	4	953	179
8	11	1006	27
9	13	1011	30
10	43	1023	120
11	15	907	184
12	76	1009	469
13	53	1020	262
14	28	1023	275
15	40	1018	43
16	10	754	114
17	5	1023	5
18	13	1020	30
19	13	1014	66
20	14	1022	69
21	1	1019	4
22	32	1023	60
23	10	1019	46
24	2	967	52
25	31	1022	207
26	92	1023	992
27	1	1001	1
28	59	1023	227
29	13	1023	30
30	2	582	2
31	2	919	2
32	5	1023	5
33	4	987	4
34	2	1023	4
35	1	652	7
36	20	1015	25
37	1	528	1
38	2	902	2
39	3	1023	3
40	14	1021	20
41	76	1023	144
42	1	964	1
43	9	1023	9
44	1	1013	1

45	1	967	1
46	2	839	2
47	4	1012	4
48	12	1023	54
49	14	1003	46
50	9	1017	100
51	23	1023	74
52	2	930	2
53	1	1022	9
54	40	1014	160
55	2	966	3
56	13	918	14
57	2	1016	2
58	1	821	1
59	18	1020	87
60	3	560	3
61	3	1001	3
62	130	1023	207
63	6	1017	6
64	2	1004	8
65	18	1012	31
66	80	1023	268
67	4	900	4
68	20	1023	86
69	4	1023	39
70	1	995	1
71	5	1022	5
72	3	741	5
73	1	962	1
74	4	986	4
75	5	1013	5
76	5	955	5
77	4	1019	4
78	4	967	4
79	3	598	3
80	11	1021	12
81	2	949	2
82	3	1020	5
83	1	816	1
84	45	1023	202
85	4	1019	7
86	0	1010	2
87	6	1014	6
88	2	1013	2
89	1	931	1
90	1	270	13

91	9	972	9
92	1	784	1
93	2	1023	2
94	2	954	2
95	2	862	2
96	4	985	9
97	1	1004	1
98	2	1016	2
99	2	619	2
100	3	967	3
101	4	1019	6
102	2	901	2
103	1	1011	1
104	4	1022	4
105	3	1010	4
106	3	1019	6
107	5	1023	35
108	38	765	83
109	1	798	1
110	1	1018	1
111	2	1020	4
112	3	623	3
113	1	959	1
114	4	1003	4
115	10	1013	10
116	2	722	2

Supplementary Table 3: Comparison of the true count of bins containing a match for a pattern vs the number of bins found by csearch and TetRex.

Index	Name	ID	Pattern	Length
1	ASN_GLYCOSYLATION	PS00001	N-{P}-[ST]-{P}	4
2	CAMP_PHOSPHO_SITE	PS00004	[RK](2)-x-[ST]	3
3	PKC_PHOSPHO_SITE	PS00005	[ST]-x-[RK]	3
4	CK2_PHOSPHO_SITE	PS00006	[ST]-x(2)-[DE]	3
5	TYR_PHOSPHO_SITE_1	PS00007	**[RK]-x(2)-[DE]-x(3)-Y**	5
6	MYRISTYL	PS00008	**G-{EDRKHPFYW}-x(2)-[STAGCN]-{P}**	5
7	AMIDATION	PS00009	x-G-[RK]-[RK]	4
8	ER_TARGET	PS00014	[KRHQSA]-[DENQ]-E-L>	4
9	RGD	PS00016	R-G-D	3
10	ATP_GTP_A	PS00017	**[AG]-x(4)-G-K-[ST]**	5
11	HISTONE_H4	PS00047	G-A-K-R-H	5
12	TUBULIN_B_AUTOREG	PS00228	<M-R-[DE]-[IL]	4
13	PRENYLATION	PS00294	C-{DENQ}-[LIVM]-x>	4
14	MICROBODIES_CTER	PS00342	[STAGCN]-[RKH]-[LIVMAFY]>	3
15	TYR_PHOSPHO_SITE_2	PS60007	**[RK]-x(3)-[DE]-x(2)-Y**	5

Supplementary Table 4: The names, IDs, and lengths of the 15 patterns estimated to be less than 6 characters in length and thereby unsuitable to be queried with TetRex. In bold are 4 patterns containing min-max quantifiers that actually are long enough to be queried.
